# Characterization of Endophytic *Streptomyces rhizosphaericola* Ahn75 and Its Potential for Biocontrol against Rice Blast

**DOI:** 10.4014/jmb.2407.07018

**Published:** 2024-10-22

**Authors:** Zujiao Fu, Rong Xiao, Zhan Hu, Min Zhang, Shandong Wu, Zhaohui Guo, Rongjun Luo, Shiping Shan, Hua Yang

**Affiliations:** National Collection of Plant-associated Microbes (Hunan), Hunan Institute of Microbiology, Changsha 410009, P.R. China

**Keywords:** Endophyte *Streptomyces*, *Magnaporthe oryzae*, genome sequence, antimicrobial compounds, greenhouse experiment

## Abstract

Plant endophyte *Streptomyces* are excellent candidates as biocontrol agents against the rice blast fungus, *Magnaporthe oryzae*. In this study, a novel strain Ahn75 with antifungal activity was isolated from healthy rice stem and identified as *Streptomyces rhizosphaericola* by phenotypic characterization and phylogenetic analysis based on 16S rRNA gene, multilocus and genome sequences. Inhibition test using culture filtrate showed that Ahn75 could effectively suppress *M. oryzae*, with mycelia growth inhibition rate of 80.88% and spore germination inhibition rate of 78.26%. Genome sequence analysis of strain Ahn75 showed 40 gene clusters of secondary metabolites and several genes related to plant growth promotion were predicted in the genome of Ahn75. Several antimicrobial compounds including valinomycin, tetrabutylammonium, and benzalkonium chloride, were also detected in the antifungal fraction from Ahn75 culture filtrate by liquid chromatography and high resolution mass spectrometry. Meanwhile, strain Ahn75 demonstrates UV tolerance under UV irradiation for 60 min, pH tolerance between pH6 and pH9, and a high halotolerance in 7% (w/v) of NaCl. Greenhouse experiments indicated that Ahn75 is able to colonize rice stems, roots, and leaves, which help rice to reduce the rice leaf blast incidence by 59.76%. All these findings suggest that strain Ahn75 could be a potential biocontrol agent for rice blast.

## Introduction

Rice blast disease caused by *Magnaporthe oryzae* (anamorph *Pyricularia oryzae*) is one of the most serious diseases in cultivated rice (*Oryza sativa* L.), which is the staple food resource for more than half of the world’s population [1]. At present, the management of rice blast mainly relies on chemical fungicides and resistant rice varieties. However, chemical fungicides not only bring serious pollution to ecology and the environment, but also have deleterious implications for human health. Meanwhile, some resistant varieties became susceptible after a few years of cultivation due to blast fungus evolution and adaptation [2]. Therefore, microbiological control has been considered as one of the most promising alternatives for the management of rice blast. Previous studies showed that rhizospheric *Bacillus amyloliquefaciens* UASBR9 [1], *Pseudomonas* sp. EA105 [3], *Trichoderma* sp.[4], and *Streptomyces* sp. [5] could inhibit the growth of the rice blast fungus. Among the microbial candidates for biocontrol, actinomycetes, especially endophytic actinomycetes, are of the utmost value because of their high antibiotic productivity, filamentous and sporulating properties, and excellent colonization aptitudes [6, 7].

Endophytic actinomycetes, which live in the inner parts of rice and do not cause any damage or disease to the host plants, can kill or inhibit pathogenic microorganisms, induce systemic resistance, or promote growth in host plant through the secretion of abundant active compounds [6]. The unique plant inner space also creates more probability of novel endophytic actinomycetes and active compounds and favors endophytes to exert beneficial effects without being influenced by field operations, climate change, and other factors for their growth [8, 9]. For example, endophyte *S. hygroscopicus* OsiSh-2 has been found to inhibit the growth of *M. oryzae* and reduce rice blast disease severity by 59.64%, which may be due to the antagonistic activity by antibiotic nigericin [10], iron competition strategy by siderophores [11], and damage to the cell wall and membrane in pathogens by lytic enzymes [7]. Endophytic *Streptomyces* sp. SS1, SS5, and SS8 colonizing rice tissues not only inhibit *M. oryzae* by the production of active metabolites and up-regulation of rice defense response genes, but also significantly enhance rice growth, with an increased wet shoot weight of 55%–518% [12]. The endophytic actinomycetes resources used as biopesticides and biofertilizers agents are still insufficient to meet the needs of rice cultivation, although some of them have showed good biocontrol and plant growth-promotion properties.

To explore novel high-efficiency biocontrol resources against rice blast, this study isolated and characterized rice endophytic actinomycetes against *M. oryzae*. An *Streptomyces* isolate with good antagonistic effects against *M. oryzae* was obtained from the stem of rice in Hainan, China. This work analyzed the antagonistic activity of this strain and evaluated its biocontrol potential in rice cultivation.

## Materials and Methods

### Isolation of Endophytic Actinomycetes

Endophytic actinomycetes were isolated from rice tissues collected from rice variety ‘Xiangliangyou 900’ in Sanya, Hainan, China. The rice plants were washed thoroughly, dried overnight, divided into root, stem, sheath and leaf tissues, cut into pieces (5 cm in length), and then subjected to a surface sterilization procedure as described by Xiong *et al*. [13].

After surface sterilization, the dried healthy and diseased plant segments were cut into pieces (1 cm in length) and transferred onto five different isolation media: humic acid vitamin B agar (HV), mannitol soybean agar (MS), tap water yeast extract agar (TWYE), water agar (WA) [10, 14, 15] and nutrient agar (NA). Each medium was pre-supplemented with 20 μg·ml^−1^ nalidixic acid and 50 μg·ml^−1^ benomyl as antibacterial and antifungal agents, respectively. Isolation plates were incubated at 27°C and 37°C and checked every 2 days. The actinomycete colonies emerged from tissues were transferred on half-strength potato dextrose agar (half-PDA) for purification.

### Screening of Antagonistic Actinomycetes against *M. oryzae*

Rice blast pathogenic fungi *M. oryzae* TJ40-2-1 were kindly provided by Zhou Hu of Hunan Agricultural University (China) [16]. The antagonistic activity of actinomycetes against *M. oryzae* was analyzed using a dual culture assay. *M. oryzae* was cultured on yeast extract-malt extract agar (ISP2) [17] for 5–7 days; then, a new 8-mm-diameter mycelia plug was cut and inoculated onto the center of a new ISP2 plate, and three actinomycetes were inoculated onto the same plate, 30 mm from the center, by sterile toothpick. There were three replicates for each strain. After incubation at 28°C for 7 days, the radius of the *M. oryzae* colony was measured. The inhibition rate of mycelia growth was determined by the following equation:

Inhibition rate = (Radius of control *M. oryzae* colony – Radius of treated *M. oryzae* colony) / (Radius of control *M. oryzae* colony – Radius of the original plug).

### Phenotypic and Phylogenetic Analysis

The cultural characteristics of Ahn75 were determined on PDA and ISP2 plate at 28°C for 5-7 days. The mycelia and spores of Ahn75 were examined by phase microscopy (Zeiss A1, Germany).

Genomic DNA was extracted using a commercial DNA extraction kit (Ezup Column Bacteria Genomic DNA Purification Kit, Sangon Biotech, China) following the manufacturer’s protocol. The 16S rRNA gene was amplified by PCR and sequenced using the universal primers 27F/765R and 705F/1492R [18]. An Illumina PE library was contructed using genomic DNA of Ahn75, and sequenced using Illumina HiSeq 2500 platform. After quality filtering, qualified reads were assembled into a single contig by SOAPdenovo v2.04; and the local cavity was filled and the base was corrected by GapCloser v1.12. Gene predictions were performed by Glimmer 3.02 (http://www.cbcb.umd.edu/software/glimmer/). rRNA and tRNA genes were predicted by Barrnap 0.4.2 and tRNAscan-SE v1.3.1, respectively. Functional annotations of predicted genes were based on BlastP similarity searches (E-value < 10^−5^) against different databases, including the NCBI Non-Redundant Protein database (Nr, http://www.ncbi.nlm.nih.gov/), the STRING database (http://string-db.org/), Clusters of Orthologous Groups database (COG, http://www.ncbi.nlm.nih.gov/COG/), Kyoto Encyclopedia of Genes and Genomes database (KEGG, http://www.kegg.jp/kegg/), and the Gene Ontology database (GO, http://www.geneontology.org/).

Taxonomical identification of Ahn75 are based on the 16S rRNA gene sequence analysis, multilocus sequence analysis (MLSA) of five housekeeping genes (*atpD*, *gyrB*, *recA*, *rpoB*, and *trpB* genes) and genome sequence analysis.

The sequences of 16S rRNA genes were used to construct the neighbor-joining phylogenetic tree by Clustal W2 and MEGA7.0. The sequences for five loci, namely, *atpD*, *gyrB*, *recA*, *rpoB*, and *trpB* genes from Ahn75 genome sequence, were concatenated head to tail in frame and exported in FASTA format [19]. The sequences of representative *Streptomyces* strains were downloaded from the GenBank database, the *Streptomyces* PubMLST database (http://pubmlst.org/streptomyces/) and the EzBioCloud database. All sequences were aligned using Clustal W2 and MEGA7.0, and a neighbor-joining phylogenetic tree was constructed from the concatenated sequences of all five loci.

Calculations of average nucleotide identity (ANI) were performed using OrthoANIu tool (www.ezbiocloud.org/)[20]. In silico DNA-DNA hybridization (DDH) values between Ahn75 and its nearest phylogenetic neighbours were calculated using Genome-to-Genome Distance Calculator 3.0 (GGDC) with the BLAST+ (recommended) method (https://ggdc.dsmz.de/ggdc.php#) [21]. As a further extension of genome-based phylogeny, the classification of Ahn75 was identified by Type (Strain) Genome Server (https://tygs.dsmz.de/).

### Antifungal Activity against *M. oryzae*

Strain Ahn75 was inoculated into ISP2 liquid medium and incubated at 28°C and 180 rpm for 7 days, and the cell-free culture was obtained by centrifugation at 12,000 g for 10 min at 4°C, then filtered through a 0.22 μm sterile membrane. The culture filtrate was diluted with sterile water; then, 1 ml stock or diluted liquid was added to 9 ml of ISP2 liquid medium at 50°C, mixed rapidly, and poured into Petri dishes (90 mm in diameter). The control comprised 1 ml sterile water in place of the culture filtrate. After the agar cooled down, a new 8-mm-diameter mycelia plug of *M. oryzae* was inoculated onto the center of the ISP2 medium containing 0, 1, 2, 5, and 10% culture filtrate of Ahn75. Each experiment was performed three times. After incubation at 28°C for 7 days, the diameter of the *M. oryzae* colony was measured. The mycelial growth inhibition rate = (Diameter of control *M. oryzae* colony – Diameter of treated *M. oryzae* colony) / (Diameter of control *M. oryzae* colony – Diameter of the original plug). And the IC_50_ was measured by SPSS v19.0. The hyphal morphology of *M. oryzae* grown on coverslips, which was inserted obliquely in the ISP2 agar medium containing culture filtrate of Ahn75, was observed using a phase contrast microscope.

For spore germination inhibition tests, spores of *M. oryzae* were harvested in sterile water by flooding the oatmeal–tomato agar plates (30 g·l^−1^ oatmeal, 150 ml·l^−1^ tomato juice, and 20 g·l^−1^ agar) of 10-day-old cultures. Then, the spore suspension was filtered through a muslin cloth to remove the mycelia, adjusted to approximately 5×10^5^ spores·ml^−1^, and used in the following bioassays. The culture filtrate of Ahn75 was added to 100 μl ISP2 liquid medium containing the spore suspension (1 × 10^5^ spores·ml^−1^) of *M. oryzae* to yield a final concentration of 5, 10, 20, and 50% culture filtrate (v/v). The ISP2 liquid medium only containing the spore suspension (1 × 10^5^ spores·ml^−1^) was used as the control. After incubation at 28°C for 16 h, the germinated and non-germinated spores in each culture were visualized and counted using phase contrast microscope. Six images were visualized on each treatment, which was repeated three times. Percentage germination was calculated by the number of germinated spores and the total number of spores in the images [3]. Spore germination inhibition rate = (Control spore germination rate – Treated spore germination rate) / (Control spore germination rate)

### Plant Growth-Promoting (PGP) Related Traits

The PGP traits of Ahn75, including nitrogen fixation, phosphate solubilization, potassium solubilization, and siderophore and IAA production, were qualitatively determined by following standard procedures. Nitrogen-free agar [6], Pikovskaya agar containing tricalcium phosphate, Aleksandrov agar used potassium feldspar powder as the only source of K, and chrome azurol-s (CAS) agar [16], were used for the evaluation of nitrogen fixation, phosphate solubilization, potassium solubilization, and siderophore production, respectively. Indole acetic acid (IAA) production was determined by the colorimetric Salkowski’s assay [16], after inocubation of Ahn75 in ISP2 for 72 h. Appearance of pink colour indicates IAA production.

### Analysis of Secondary Metabolites

Secondary metabolite gene clusters on strain Ahn75 genome sequence were predicted by antiSMASH v5.0.0 (http://antismash.secondarymetabolites.org/). Secondary metabolites with antifungal activity were prepared from the culture filtrate of strain Ahn75, which was extracted with an equal volume of N-butanol twice and evaporated to dryness using rotary evaporator at 40°C. Then, the crude extracts were suspended into sterile water and filtered through a 0.22 μm membrane to be subjected to antifungal assays against *M. oryzae*. Then active fraction was injected onto Poroshell 120 Hilic-Z column (4.6 × 150 mm, 2.7 μm; Agilent, USA) that was coupled in-line with an LTQ-Orbitrap XL mass spectrometer (Thermo Fisher Scientific, USA). The LC gradient was run for 22 min from 100% to 50% of acetonitrile at flow rate 400 μl·min^−1^. Mass spectrum were analyzed using Global Natural Products Social (GNPS) Molecular Networking [22].

### Abiotic Stress Resistance Assay

Strain Ahn75 was incubated on ISP2 plate at 28°C 5 days, and the spores were harvested in sterile water with 0.05% Tween 80, and diluted to a concentration of approximately 1 × 10^8^ cfu·ml^−1^.

UV tolerance was performed by spreading 5 ml spore suspension of strain Ahn75 on plates with a diameter of 9 cm and were placed 30 cm below the ultraviolet light (wavelength 254 nm) [23]. The irradiation lasts for 0 min, 10 min, 30 min, and 60 min respectively. After irradiation, tenfold serial dilutions of each samples were spread on ISP2 agar medium plate in twice and incubated at 28°C for 2 days. The number of colony forming units (CFU) in the agar plates were counted as survival spores. This experiment was repeated triplicate independently, and the means of three replicates was used as the survival number of Ahn75 spores after UV irradiation.

pH tolerance was determined by inoculating 1 ml of spore suspension of strain Ahn75 into 500-ml flasks containing 50 ml of fresh ISP2 medium adjusted to pH values 4–9 and buffered with phosphate. The culture was incubated in triplicate replicates at 28°C and 180 rpm for 7 days. Then the cells of strain Ahn75 were harvested by centrifugation at 4,000 g for 10 min, and weighted as the wet biomass, the supernatant filtered by 0.22 μm membrane was used for antifungal assay against *M. oryzae*.

Halotolerance was carried out by inoculating 2 μl of spore suspensions of strain Ahn75 in 96-well microtiter plates, each of which contained 200 μl of ISP2 medium amended, respectively, with 0%, 1%, 3%, 5%, 7%, and 9%(w/v) of NaCl [24]. The culture was then incubated in five replicates at 28°C and 500 rpm for 32 h. The growth curve was monitored every four hours using a spectrophotometer (Thermo Fisher Scientific) at (λ) 600 nm. Non-inoculated ISP2 medium with the above salt concentrations was used as a blank.

### Greenhouse Experiment

The colonization ability and biocontrol effects of strain Ahn75 were investigated on rice variety ‘Xiangliangyou 900’ in the greenhouse. Rice seeds were soaked in sterile distilled water for 30 min, and healthy seeds were selected, surface sterilized with 75% ethanol for 5 min, washed five times with sterile distilled water, and transferred to a double layer drain basket. Seeds were germinated in an artificial climate incubator at 25°C for 1 weeks. Part seedlings were transferred into plastic box and grown in sterile Hoagland solution [25] with a photoperiod of 12 h/ 12 h (light/darkness). Until 2–3 leaf stage, the rice seedlings was sprayed with Ahn75 spore suspension. Colonization of strain Ahn75 in rice tissue, including root, stem and leaf was recorded on ISP2 medium cultivated with surface-sterilized and ground tissue extracts by viable count at 1-27 days after inoculation.

After root-soaking in an Ahn75 spore suspension (containing 10^8^ spores·ml^−1^ in 0.05% v/v Tween 80) for 1 h, seedlings were transplanted to plastic pots (30 cm × 18 cm × 20 cm) filled with 4.0 kg field soil and 0.5 kg vermiculite, with 3 seedlings per pot. Rice plants were routinely grown in the greenhouse with natural sunlight, and reinoculated by spraying with an Ahn75 spore suspension at the tillering stage. A control assay was conducted simultaneously, using sterile water in place of Ahn75 spore suspension. The experiment was repeated three times. Then, all treated plants were sprayed with *M. oryzae* conidial suspension (10^4^ spores·ml^−1^) 5 days after the second Ahn75 spray application. The disease lesions on the leaf surface were counted 10 days after pathogen infection, and the leaf blast incidence was calculated by dividing the number of diseased plants by the total plants in each pot. And the grains of each pot were collected, dried and analyzed at maturity phase of rice.

### Statistical Analysis

Data were expressed as means ± standard error of the mean (SEM). The differences in the means of all results were examined by one-way ANOVA with Dunnett’s multiple comparison post-hoc tests using SPSS v19.0. Differences were considered significant when *p* < 0.05.

### Genome Sequence Accession number

The draft genome sequence of strain Ahn75 was deposited at GenBank under accession number JAJQWZ000000000.

## Results

### Isolation and Screening of Antifungal Endophytic Actinomycetes

In this study, 122 endophytic actinomycete strains were isolated from healthy and diseased rice segments. Among them, 65.57% strains showed antagonistic activity against *M. oryzae*, and the frequency of antagonistic endophytes was associated with the tissue source, culture medium, and temperature by statistical analysis (Table S1). More antagonistic actinomycetes appeared from healthy rice stems, and on the TWYE and MS media at 37°C. One isolate, Ahn75 (CCTCC No. M 2019890), which was obtained from a healthy stem on TWYE medium at 37°C, showed an inhibition rate of 53.49% ± 3.38% against *M. oryzae* by dual culture assay.

### Phenotypic Analysis of Ahn75

**Fig. 1:**
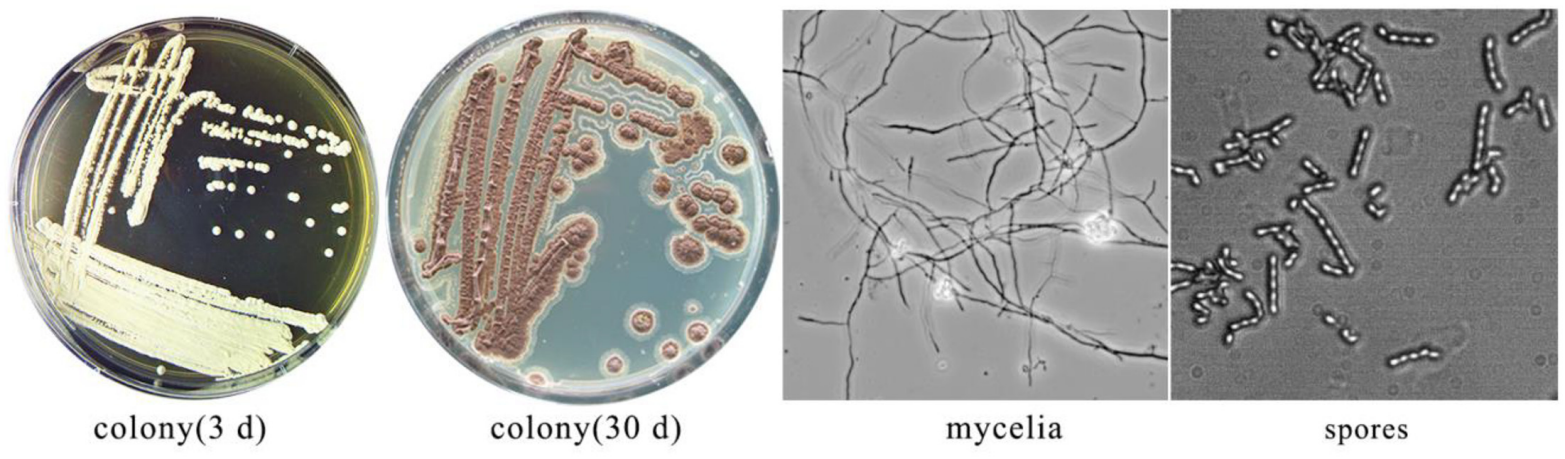
Colony and cell morphology of strain Ahn75 cultured in ISP2 media.

Strain Ahn75 grew on different agar media. However, it grew better on ISP2 medium than PDA medium. When it was grown on ISP2 agar medium, the colonies were round with white aerial mycelium and gray-green spores after 2–3 d. The substrate mycelium was white at early cultivation and then turned brown gradually (Fig. 1).

### 16S rRNA Sequence Analysis and MLSA of Ahn75

**Fig. 2:**
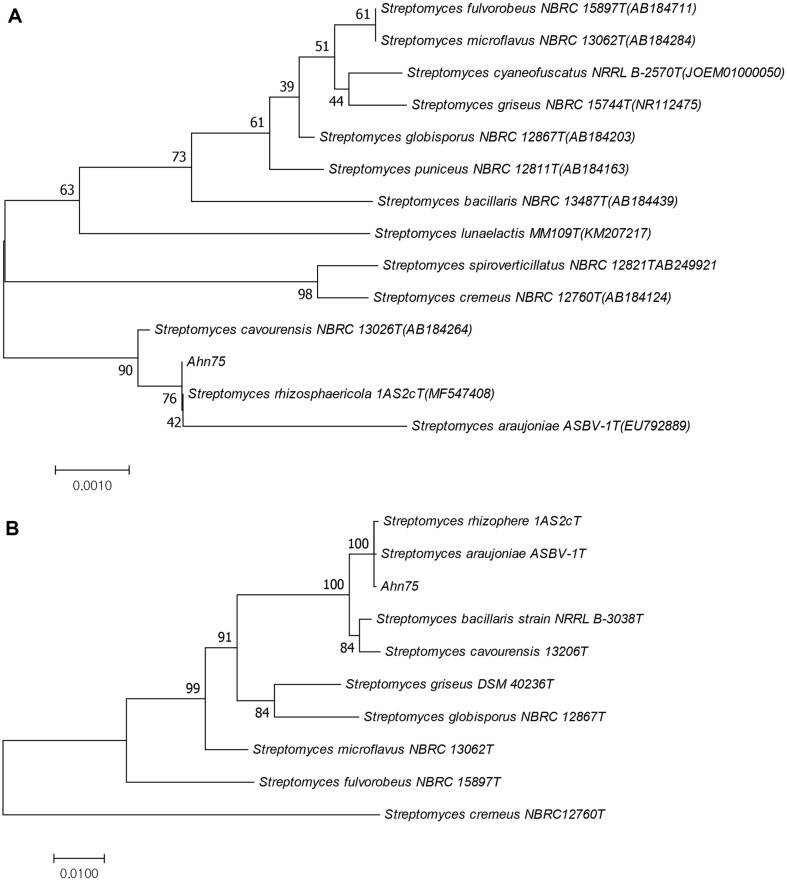
Neighbor-joining trees based on 16S rRNA sequences (A) and MLSA from five loci (*atpD*, *gyrB*, *recA*, *rpoB*, and *trpB*) (B) of strain Ahn75. The tree was drawn to scale, with branch lengths in the same units as those of the evolutionary distances used to infer the phylogenetic tree. The evolutionary distances were computed using the maximum composite likelihood method and are in the units of the number of base substitutions per site. Nucleotide sequences of 10 reference strains in Fig. 2A and 2B came from the GenBank database and the *Streptomyces* PubMLST database, respectively. All positions containing gaps and missing data were eliminated. Evolutionary analyses were conducted in MEGA7.0.

From 16S rRNA gene sequence analysis using BLAST at NCBI, the strain was classified as the genus *Streptomyces*. Both the phylogenetic trees based on 16S rRNA and MLSA of five housekeeping genes (*atpD*, *gyrB*, *recA*, *rpoB*, and *trpB*) showed that strain Ahn75 had the closest sequence similarity with type strain *S. rhizosphaericola* 1AS2c and *S. araujoniae* ASBV-1 (Fig. 2A and 2B). Strain Ahn75 shared 16S rRNA sequence similarity of 99.70-100% with *S. araujoniae* ASBV-1, *S. cavourensis* NBRC 13026, and *S. rhizosphaericola* 1AS2, and an MLSA distance of 0.0008 with *S. rhizosphaericola* 1AS2c and *S. araujoniae* ASBV-1, 0.0092 with *S. bacillaris* NRRL B-3038, and 0.0126 with *S. cavourensis* NBRC 13026. The evolutionary distances between Ahn75 and *S. rhizosphaericola* 1AS2c, *S. araujoniae* ASBV-1 were below the species definitive MLSA distance of 0.007, and the evolutionary distance between *S. rhizosphaericola* 1AS2c and *S. araujoniae* ASBV-1 based on five housekeeping genes (*atpD*, *gyrB*, *recA*, *rpoB*, and *trpB*), was also below 0.007, which is different from the results of 0.012 between the two strains based on six genes (16S rRNA, *atpD*, *gyrB*, *recA*, *rpoB*, and *trpB*) by Vargas Hoyos *et al*. [26], which maybe caused by the difference length of choosed gene sequences.

### Genome Analysis of Ahn75

**Fig. 3:**
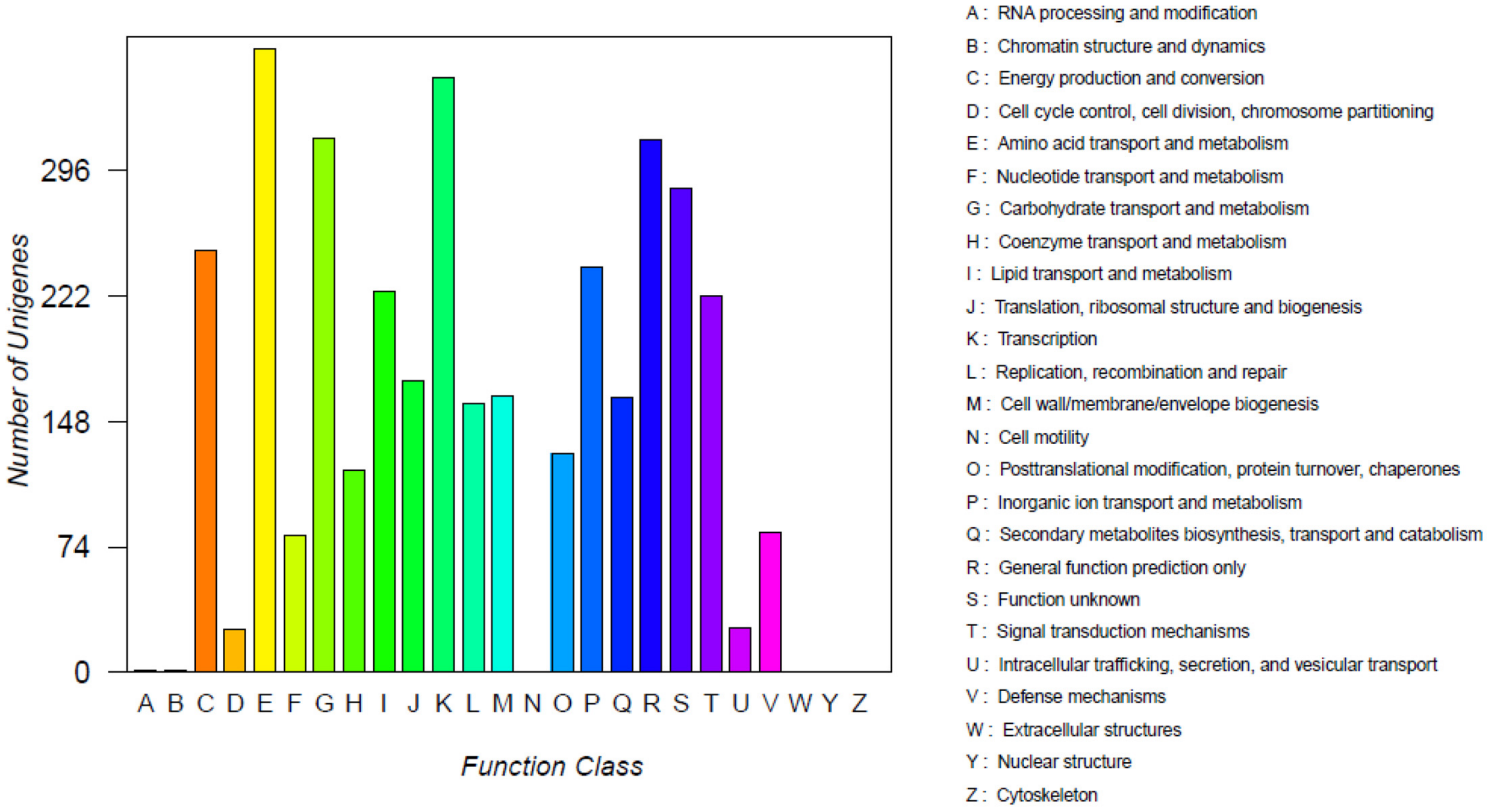
Function class of strain Ahn75 by Clusters of Orthologous Groups database (COG) function classification.

In order to determine the taxonomy of strain Ahn75, its genome was sequenced using Illumina HiSeq 2500 platform. The draft genome of strain Ahn75, which was deposited at GenBank under accession number JAJQWZ000000000, had a consensus length of 7,550,402 bp assembled by 115 scaffolds. Gene predictions resulted in 6,980 open reading frames (ORFs). The function of 6,435 ORFs was annotated and 3,556 ORFs were classified into 21 COG categories by their function. Among the 21 COG categories, the cluster for “amino acid transport and metabolism” represented the largest group (368, 9.97%), followed by “transcription” (351, 9.51%) and “general function prediction” (314, 8.51%) (Fig. 3). The genomic DNA G+C content of strain Ahn75 was 72.32 mol%. The ANIu between Ahn75 and the type strain of the most closely related species, *S. rhizosphaericola* 1AS2c, *S. bacillaris* NRRL B-3038, and *S. cavourensis* NBRC 13026 were 99.21%, 95.01% and 94.72%, respectively. The DDH value of Ahn75 and *S. rhizosphaericola* 1AS2c was 93.80%, which was much higher than the DDH of Ahn75–*S. bacillaris* NRRL B-3038 (60.70%), Ahn75–*S. cavourensis* NBRC 13026 (59.10%), and the recommended threshold for species delineation (Table S2). The analysis by Type (Strain) Genome Server also showed Ahn75 belongs to known species *S. rhizosphaericola* (Table S3). Thus, all the results based on phylogenomic analysis of 16S rRNA, MLSA, and genomic comparism and phenotypic properties suggest that strain Ahn75 should be considered the species *S. rhizosphaericola*.

### Activity of Strain Ahn75 against *M. oryzae*

**Fig. 4:**
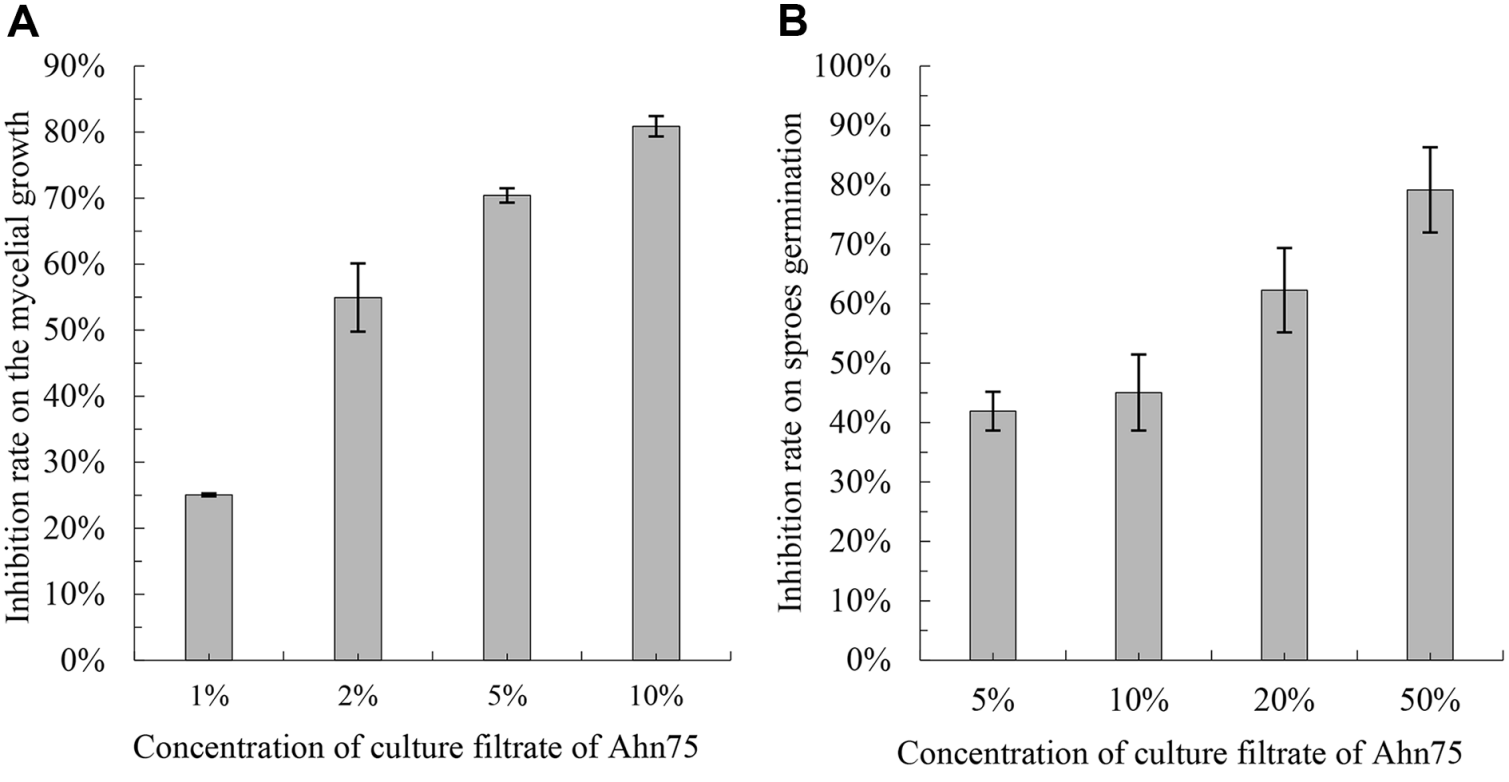
Inhibition of the culture filtrate of Ahn75 on the mycelial growth and spore germination of *Magnaporthe oryzae*. (**A**) Inhibition rate on the mycelial growth of *M. oryzae*; mean ± SD, *n* = 3. (**B**) Inhibition rate on the spore germination of *M. oryzae*; mean ± SD, *n* = 3.

The mycelial growth of *M. oryzae* on ISP2 agar medium containing culture filtrate of Ahn75 was inhibited, and the inhibition rate was dependent on the concentration of the culture filtrate (Fig. 4A). When the concentration of the culture filtrate ranged from 1% to 10%, the inhibition rate of mycelial growth gradually increased from 25.05%to 80.88% with an IC_50_ value of 2.21% (95% confidence interval, 0.522% to 4.645%).

Meanwhile, the spore germination of *M. oryzae* was also be inhibited by the culture filtrate of Ahn75 (Fig. 4B). When 50% culture filtrate by volume was used, the inhibition rate of spore germination reached 79.15% ± 7.18.

**Fig. 5:**
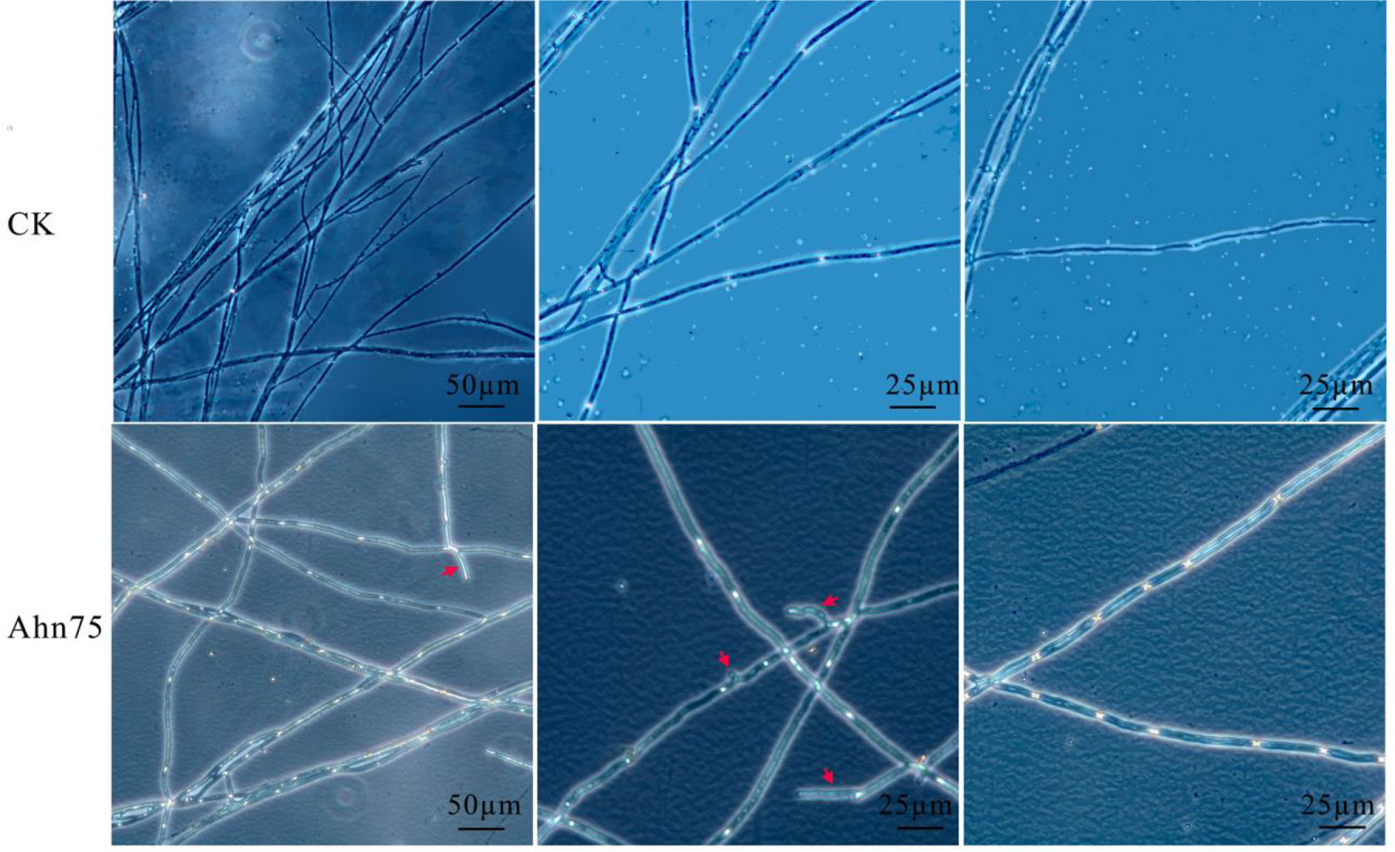
Mycelial morphology of *Magnaporthe oryzae* treated with a Ahn75 culture filtrate. CK: *Magnaporthe oryzae* grown on ISP2 medium; Ahn75: *Magnaporthe oryzae* grown on ISP2 medium with 5% Ahn75 cultural filtrate. The mycelial was observed in phase microscopy, and the the arrow indicates the deformed mycelial.

Using a phase contrast microscope, it was observed that the mycelia of *M. oryzae* became shorter when treated with 2% and 5% culture filtrate of Ahn75, as did the single cell, and part of the cells appeared swollen, distorted, or formed vesicle structures (Fig. 5).

These results indicated that the culture filtrate of Ahn75 can significantly inhibit mycelial growth and spore germination of *M. oryzae* with concentration dependence, and cause mycelia to be shorter, swollen, or distorted.

### Identification of Antifungal Compounds

Through genome mining by the antiSMASH tool, 40 candidate secondary metabolite clusters were predicted in the Ahn75 genome, including three lactones, seven non-ribosomal peptide synthetases (NRPS) (valinomycin, salinomycin, griseoviridin, malacidin, phosphonoglycans and daptomycin), two Nrps-like, three Nrps-pks (diisonitrile antibiotic SF2768, cosmomycin D, and SGR PTMs.), four siderophores (desferrioxamine B, griseobactin, coelichelin, and ficellomycin), eight polyketide antibiotics (bafilomycins, salinomycin, brasilinolide, nonactin, auricin, alkylresorcinol, and herboxidiene), five terpenes (geosmin, hopene, isorenieratene, and stambomycin), six peptides, and two others (Table S4). The total lengths of these gene clusters were estimated to be about 1,196 kb, which suggested that 15.84% of the genome may be occupied by genes concerned with the biosynthesis of secondary metabolites, a far higher proportion than that found in other sequenced genomes.

**Fig. 6:**
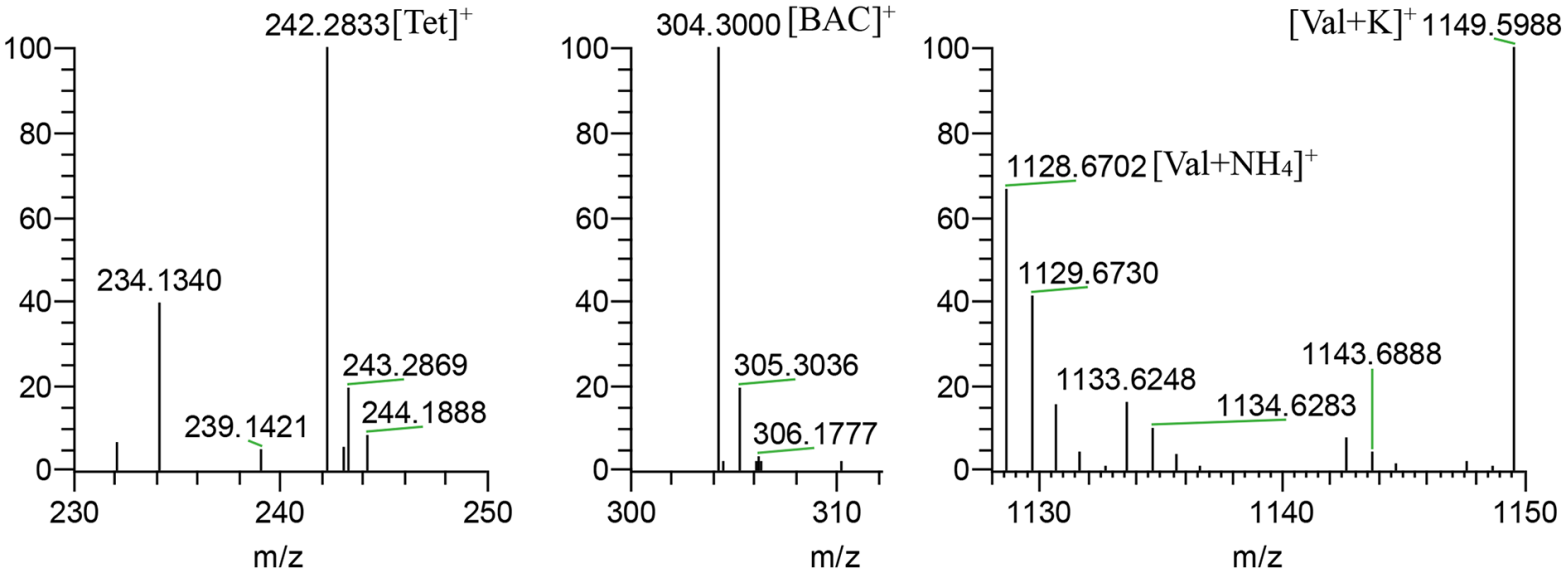
Mass spectrum of compounds synthesized by Ahn75. Ions of m/z 242.2833 and 304.3000 corresponded to tetrabutylammonium [Tet]^+^ and benzalkonium chloride (C12) [BAC]^+^, respectively. Ions of m/z 1128.6702 and 1149.5988 corresponded to valinomycin [Val + NH_4_]^+^ and [Val+K]^+^ , respectively.

In addition, three compounds: valinomycin, tetrabutylammonium, and benzalkonium chloride were successfully detected from the active extracts of Ahn75 through MS1 data (Fig. 6) and MS2 data (Fig. . S1A-S1D). Ions of m/z values 1,128.6702 and 1,149.5988 corresponded to valinomycin [M + NH_4_]^+^ (calcd for C_54_H_94_N_7_O_18_^+^, 1,128.6655) and [M+K]^+^ (calcd for C_54_H_90_N_6_O_18_K, 1,149.6), respectively [27, 28]. And ions of m/z values 242.2833 and 304.3000 corresponded to tetrabutylammonium [Tet]^+^ (calcd. for C_16_H_36_N^+^, 242.28) and benzalkonium chloride (C12) [BAC]^+^ (calcd. for C_21_H_38_N^+^, 304.29) in GNPS MASST database (Fig. 6). These compounds may play important role in the antifungal activity of strain Ahn75 against *M. oryzae*.

### Plant Growth Promotion Characteristics

Strain Ahn75 can grow on Nitrogen-free medium and Aleksandrov agar, which indicated the strain had the capacity to fix nitrogen and solubilize potassium. The qualitative determination also showed strain Ahn75 generated an orange ring on the CAS plate, and a pink color after reaction with Salkowski reagent, which indicated that Ahn75 can produce siderophore and IAA. No clear zone was observed around strain Ahn75 on Pikovskaya medium, which indicated that the strain could not dissolve phosphate.

Ahn75 genome also contains a series of genes/gene clusters associated with plant growth promotion, including siderophore biosynthesis, nitrogen fixation, phosphate and potassium transport, growth-promoting hormones (IAA, phytase, trehalose), and spermidine (Table S5). Siderophore can not only inhibit plant pathogens but also promote plant growth by providing iron to the plant. In addition, five nitrogen utilization related genes, *nifU* coding for the nitrogen fixation protein, *glnB* coding for nitrogen regulatory protein II, *moeA* and *moaD* coding for molybdenum cofactor biosynthesis protein, *nir* coding for nitrite reductase, four phosphate transporter genes *pstA*, *pstB*, *pstC*, and *pstS*, and three potassium transporter genes *trkA*, *ktrB*, and *kdp*FABC were found in the Ahn75 genome. IAA synthesis related genes *ysnE* coding for N-acetyltransferase, *dhaS* coding for indol 3-acet-aldehyde dehydrogenase, *trpC* coding for indole-3-glycerol-phosphate synthase, *yhcX* coding for nitrilase, the phytase synthesis gene *phy*, trehalose synthesis-related genes, *tpsA* coding for alpha, alpha-trehalose-phosphate synthase, *tre*PP coding for trehalose 6-phosphate phosphorylase, and *tre*Z coding for malto-oligosyltrehalose trehalohydrolase were also identified in the Ahn75 genome. The genome also contains spermidine synthase gene *spe*E, and agmatinase gene *spe*B, which are also involved in plant growth and shoot differentiation [29].

These growth promotion traits and the abundant genetic basis indicate that strain Ahn75 has great potential as a growth-promotion agent in rice cultivation.

### Abiotic Stress Resistance

**Fig. 7:**
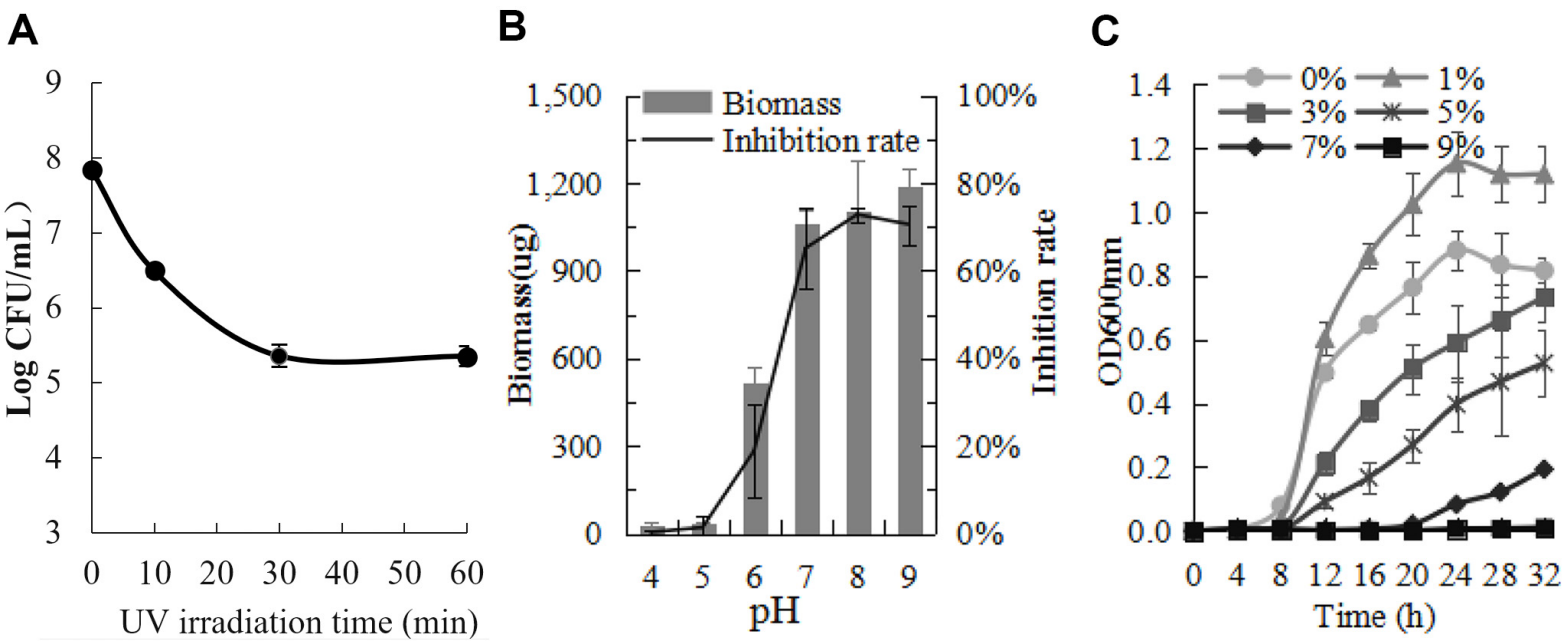
Survivor curve of strain Ahn75 cultured in ISP2, after UV irradiation (A), and with different pH (B), different salt (C).

UV resistance experiment (Fig. 7A) showed the spore germination number of strain Ahn75 after UV treatment was significantly decreased (*p* < 0.05), compared with the untreated strain (6.97 ± 0.68 × 10^7^ cfu·ml^−1^). The spore germination number of strain Ahn75 decreased slowly, as the UV irradiation time increased, but it still have 2.27±0.81×10^5^ cfu·ml^−1^ alive cells after UV irradiation for 60 min. These results indicated that spores of strain Ahn75 had a certain resistance to UV light.

Strain Ahn75 could grow on ISP2 medium with pH values between 6 and 9 (Fig. 7B). Significant growth inhibition of strain Ahn75 was observed at pH of 4, 5 and 6, but no significant difference on the mycelia biomass and inhibition rate of culture filtrate appeared between pH 7, 8 and 9 (*p* > 0.05). Highest biomass with 23.81 ± 1.16 μg/ml was obtained at pH 9, and highest inhibition rate with 72.83% ± 1.72 was obtained at pH 8.

Salt tolerance assay demonstrated Ahn75 could grow in ISP2 with the concentration of 0%-7% (w/v) NaCl while no any growth occured in 9% NaCl within 32 h after incubation (Fig. 7C). All the exponential phase between 0%and 5% were recorded after 8 h of incubation, while 7% was recorded after the 20th hour, and a clear decrease in the Optical Density (OD) of the strain was observed, as the salt concentration increased from 1% to 7% (w/v) NaCl. These results indicated Ahn75 had a higher salt tolerance.

### Biocontrol and Plant Growth Promotion Efficacy of Strain Ahn75

The green house experiments showed strain Ahn75 could colonize in the leaf, root and stem of rice seedlings for more than 3 weeks after inoculation. The colonization density of Ahn75 was increased rapidly after inoculation, and attained the highest level after 5 days, then decreased into a stable condition, which still keep 851.06, 990.59, 275.94 CFU·g^−1^ in root, stem and leaf at the 21th day after inoculation, respectively.

**Fig. 8:**
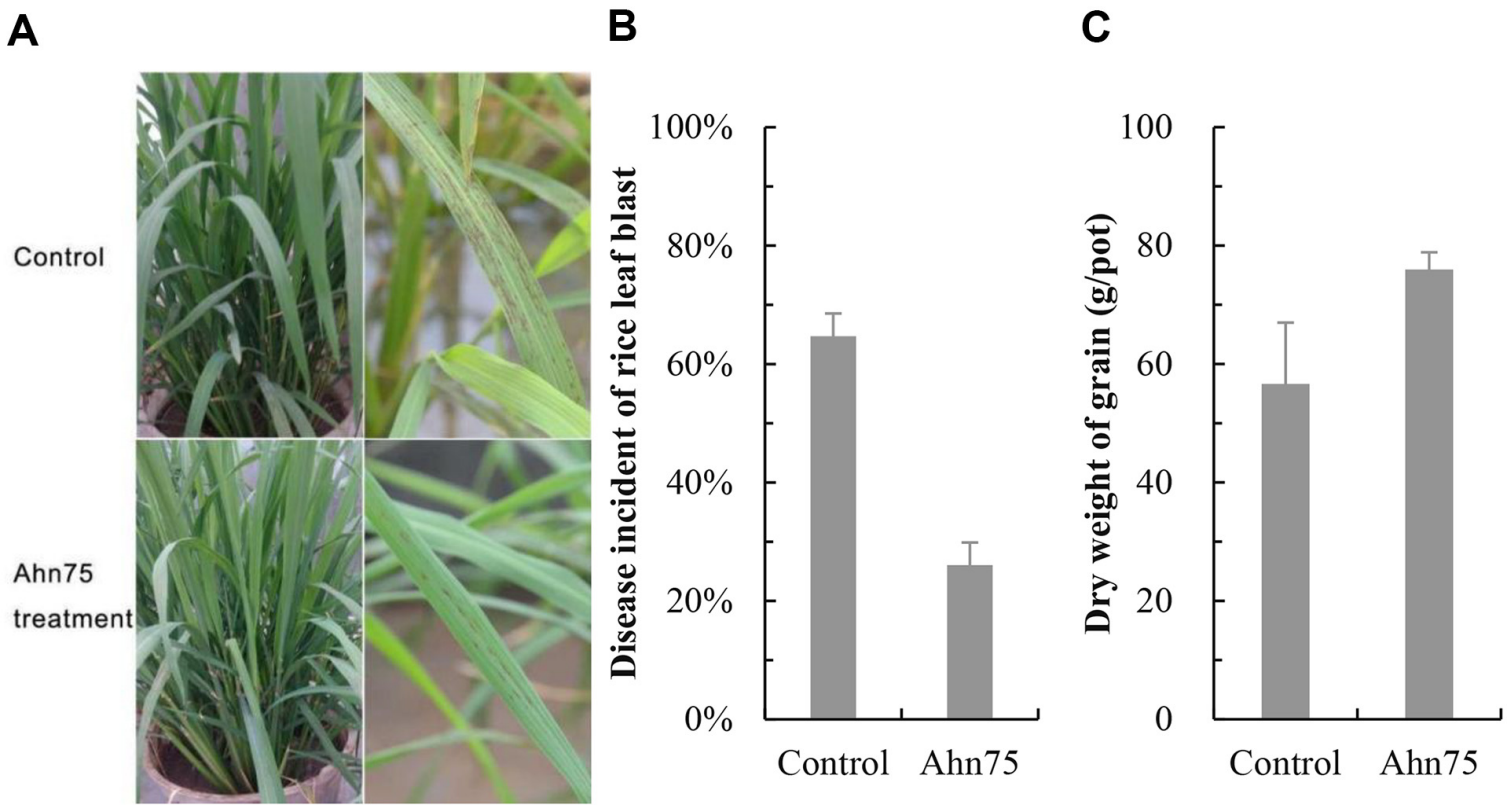
Effects of Ahn75 on rice leaf blast incidence (A, B) and grain weight (C) in pot experiment.

The leaf blast disease symptoms detected at the tillering stage showed that leaves treated with Ahn75 developed significantly less lesions than the control leaves treated with water, and an decrease in disease incident of leaf blast was also observed, with an average of 26.04% ± 7.49 for plants treated with Ahn75, as compared to the control with 64.17% ± 3.83 of disease incident (Fig. 8A and 8B). Grain yield detection during the mature phase also showed the dry weight of grains in the treatment potted rice was increased by 34.12% ± 5.13 than that in the control potted rice (Fig. 8C). The difference in disease incidence and grain weight between treated and control plants was significant (*p* < 0.05), which suggests that Ahn75 can protect rice plants against leaf blast by reducing disease incidence.

## Discussion

Plant endophyte actinomycetes were confirmed as new important resources for plant biological control and growth promotion. In this study, antifungal strains were isolated from healthy and diseased rice tissues using five media with different nutrient levels, which were incubated at 27°C and 37°C. It showed that a higher isolation frequency of antifungal actinomycetes were obtained from healthy tissues with few tiny lesions than diseased tissues with severe lesions. Few infected pathogenic fungi can stimulate the colonization and growth of antifungal microorganisms in the plant and improve their predominance in the endophyte microbial community. Moderate nutrient media, such as TWYE and MS, and a higher culture temperature, can also improve the isolation frequency of antifungal actinomycetes.

The anti-fungal strain Ahn75 was obtained and identified as *S. rhizosphaericola* based on the results of 16S rRNA, MLSA, genomic comparism, and cultural and morphological characteristics. Ahn75 and its culture filtrate could strongly inhibited the mycelia growth and spore germination of *M. oryzae* in vitro. And the in vivo greenhouse pot assay also demonstrated that the strain can protect rice against leaf blast by reducing the area of the lesion and disease incidence. The high-efficiency antifungal activity of strain Ahn75 may be due to the following three factors. One is the abundant metabolites produced by Ahn75, including siderophore, valinomycin, tetrabutylammonium, and benzalkonium chloride. Siderophore is one of the most vital antifungal metabolites from Ahn75 and was identified by the CAS assay and the search for gene clusters on the genome sequence. Zeng *et al*. [11] reported that siderophores can inhibit the growth of *M. oryzae* by competing for iron. Valinomycin is another active compound that was found in the culture filtrate from Ahn75 by LC-HRMS/MS and genome sequence, which has been reported to be a macrolactone antibiotic with a broad range of bioactivities, including antifungal, antibacterial, antiviral, insecticidal-nematocidal, and cytotoxic/anticancer activities [30]. In this paper, we also determined that valinomycin could also significantly inhibit the mycelia growth of *M. oryzae* by 52.27 ± 4.55%, when it was at a concentration of 15 μg·ml^-1^. Then, tetrabutylammonium and benzalkonium chloride were also considered disinfectants, which showed good antibacterial activity against both gram-positive bacteria (*Staphylococcus epidermidis*) and gram-negative bacteria (*Escherichia coli*) [31, 32]. In addition, the genome of Ahn75 was predicted to contain more than 30 other gene clusters for the biosynthesis of antimicrobial compounds, such as bacteriocins, lactones, Nrps, and polyketide antibiotics [33-35]. One of the T1pks, bafilomycins, which were proved to be active against *P. oryzae* (*M. oryzae*) [36], were also predicted to be present in the genome of Ahn75 and showed 83% and 38% sequence similarity with that of bafilomycin in the MIBiG BGC database (BGC0000028). The presence of abundant secondary metabolites provides very important basis for the antifungal activity of Ahn75.

The another important way in which Ahn75 protects rice against leaf blast may be due to its excellent colonization capacity, which can colonize in the stems, roots and leaves of rice seedlings for a long time after inoculation, then provide a long-term protection against rice disease.

Furthermore, environmental stress tolerance is one of the most important limiting for the application of agricultural microorganisms. In this study, Ahn75 demonstrates an ability to grow up to 7% NaCl, and its highest OD density at exponential phase appeared at 24 h in 1% NaCl, which indicated that 1% salt concentration might be more convenient to the cell proliferation of Ahn75. High salt, UV, and pH tolerance of Ahn75 may help it to survive longer in the soil and on the surface of rice plant, then provide more opportunities to colonize inside rice plant.

In addition, Ahn75 also showed several plant growth-promoting properties, including nitrogen fixation, potassium solubilization, siderophore and IAA production. Many genes/gene clusters associated with these properties were identified in the Ahn75 genome, including siderophore synthesis, nitrogen fixation, potassium transport, and IAA synthesis.

The results here indicated that Ahn75 has great potential in the biocontrol of rice blast and improving yield in cultivated rice. However, the effects of Ahn75 as a biocontrol against rice blast and growth promotor in the field deserve deeper investigation because the effects are correlated with the colonization level of Ahn75, which was influenced by the forms and quantity of beneficial microorganisms, weather conditions, and inoculated rice growth period.

## Supplemental Materials

Supplementary data for this paper are available on-line only at http://jmb.or.kr.
