# Supplementary material for: Characterization of Endophytic Streptomyces rhizosphaericola Ahn75 and Its Potential for Biocontrol against Rice Blast
Source: J Microbiol Biotechnol. 2024 Oct 22;34(12):2516–26. doi: 10.4014/jmb.2407.07018 (PMC11729360; doi:10.4014/jmb.2407.07018)
Supplement: Supplementary file 1 [file jmb-34-12-2516-supple.pdf]

J. Microbiol. Biotechnol. 2024. 34(12): 2516–2526
https://doi.org/10.4014/jmb.2407.07018

Supplementary Tables and Figure

Characterization of endophytic *Streptomyces rhizosphaericola* Ahn75 and its potential for biocontrol against rice blast

Zujiao Fu, Rong Xiao, Zhan Hu, Min Zhang, Shandong Wu, Zhaohui Guo, Rongjun Luo, Shiping Shan, Hua Yang*

Hunan Institute of Microbiology, Changsha, 410009, PR China

***Corresponding author:** dyyhua@163.com

Table S1. Separation conditions and inhibition rate of 80 rice-original antifungal actinomycetes against *Magnaporthe oryzae*.

Strain name	Isolated medium	Culture temperature (°C)	Original tissue	Inhibition rate against *M.oryzae* (means ± SEM)
Ahn1	MS	27	healthy stem	55.90%±8.02
Ahn2	MS	27	healthy stem	43.47%±3.92
Ahn3	MS	27	healthy stem	43.06%±3.92
Ahn4	MS	27	healthy stem	30.95%±1.45
Ahn5	MS	27	healthy stem	18.22%±1.82
Ahn6	MS	27	healthy stem	22.51%±2.80
Ahn7	MS	27	healthy stem	15.79%±1.05
Ahn8	MS	27	healthy stem	30.78%±0.73
Ahn10	MS	27	healthy stem	46.00%±1.46
Ahn11	MS	27	diseased stem	20.82%±1.59
Ahn13	TWYE	27	healthy stem	18.36%±1.10
Ahn14	TWYE	27	healthy stem	30.58%±2.47
Ahn18	TWYE	27	healthy stem	28.66%±9.26
Ahn20	TWYE	27	healthy stem	21.57%±0.69
Ahn21	TWYE	27	healthy root	23.69%±5.71
Ahn25	NA	27	healthy stem	45.10%±0.91
Ahn26	NA	27	healthy stem	51.46%±5.25
Ahn29	NA	27	healthy stem	6.72%±1.09
Ahn30	TWYE	37	healthy stem	62.50%±1.90
Ahn32	TWYE	37	healthy stem	8.03%±0.60
Ahn33	TWYE	37	healthy stem	8.07%±0.26
Ahn35	TWYE	37	healthy stem	7.76%±0.40
Ahn36	TWYE	37	healthy stem	54.45%±4.07
Ahn37	TWYE	37	healthy stem	16.93%±3.16
Ahn43	NA	37	healthy stem	7.34%±2.25
Ahn44	NA	37	healthy stem	8.03%±0.66
Ahn45	NA	37	healthy stem	7.57%±1.93
Ahn46	NA	37	healthy stem	17.30%±1.66
Ahn47	NA	37	healthy stem	8.56%±1.01
Ahn49	TWYE	37	diseased root	2.50%±0.68
Ahn50	TWYE	37	diseased root	2.35%±0.59
Ahn51	NA	37	diseased root	2.66%±0.81
Ahn52	TWYE	37	diseased root	4.78%±1.00
Ahn53	NA	37	diseased root	8.89%±1.50
Ahn54	TWYE	37	diseased stem	3.63%±1.88
Ahn55	WA	37	diseased stem	51.72%±2.49
Ahn56	MS	37	diseased root	51.03%±1.39
Ahn60	NA	27	diseased stem	51.83%±3.58
Ahn61	NA	27	diseased stem	4.70%±1.15
Ahn63	WA	27	healthy stem	34.63%±1.04
Ahn64	WA	27	healthy stem	7.11%±0.70
Ahn65	NA	27	healthy stem	51.89%±8.89
Ahn66	NA	27	diseased stem	48.18%±10.73%
Ahn67	NA	27	diseased stem	44.82%±7.37%
Ahn68	NA	27	diseased root	12.41%±1.87%

Ahn70	WA	27	diseased stem	45.20%±2.65%
Ahn71	MS	37	healthy stem	22.52%±2.41%
Ahn73	NA	37	healthy stem	32.23%±21.78%
Ahn74	MS	37	diseased stem	48.06%±9.18%
Ahn75	TWYE	37	healthy stem	53.49%±3.38%
Ahn76	WA	37	diseased stem	49.17%±5.19%
Ahn82	TWYE	37	healthy stem	55.94%±8.30%
Ahn87	TWYE	37	healthy stem	48.77%±1.08%
Ahn88	MS	37	healthy stem	53.89%±3.87%
Ahn91	WA	37	healthy root	26.06%±1.36%
Ahn93	NA	37	healthy root	42.25%±1.71%
Ahn94	MS	37	healthy root	7.69%±1.07%
Ahn95	TWYE	37	healthy root	2.20%±0.47%
Ahn98	TWYE	37	healthy root	17.51%±0.74%
Ahn100	TWYE	37	healthy root	27.20%±1.33%
Ahn101	TWYE	37	diseased root	15.20%±6.18%
Ahn102	MS	27	healthy stem	45.37%±1.07%
Ahn103	TWYE	27	healthy stem	40.77%±1.57%
Ahn104	MS	37	healthy stem	45.77%±2.47%
Ahn105	TWYE	37	healthy stem	41.47%±7.52%
Ahn106	NA	37	diseased stem	12.52%±1.93%
Ahn107	WA	37	diseased stem	30.82%±0.62%
Ahn109	MS	37	healthy root	54.04%±3.28%
Ahn110	MS	37	healthy root	42.77%±1.73%
Ahn112	TWYE	37	diseased root	16.43%±3.60%
Ahn113	TWYE	37	healthy leaf	36.38%±0.27%
Ahn114	HV	37	diseased leaf	40.53%±3.23%
Ahn115	HV	37	diseased leaf	38.84%±1.92%
Ahn116	HV	37	diseased leaf	37.15%±2.01%
Ahn117	HV	37	diseased leaf	40.07%±2.62%
Ahn118	HV	37	healthy leaf	37.77%±2.08%
Ahn119	MS	27	diseased stem	40.07%±2.54%
Ahn120	MS	37	diseased stem	26.49%±0.99%
Ahn122	TWYE	27	healthy stem	42.81%±0.69%

MS, mannitol soybean agar, TWYE, tap water yeast extract agar, NA, nutrient agar, HV, humic acid vitamin B agar, WA, water agar. Inhibition rate against *M.oryzae* was calculated by (Radius of control *M. oryzae* colony - Radius of treated *M. oryzae colony) /* (Radius of control *M. oryzae* colony - Radius of the original plug). Data were expressed as means ± standard error of the mean (SEM) with three separate experiments.

Table S2. Comparative analysis of 16S rRNA and genome between Ahn75 and the closest strains.

Closest strain	Similarity of 16S rRNA (%)	ANIu of Genome (%)	DDH of Genome (%)
S. rhizosphaericola 1AS2c(T)	99.63	99.21	93.80
S. cavourensis NBRC 13026(T)	99.93	94.72	59.10
S. bacillaris NBRC 13487(T)	99.33	95.01	60.70
S. puniceus NBRC 12811(T)	99.33	86.11	31.80
S. lunaelactis MM109(T)	99.33	80.05	24.20
S. globisporus NBRC 12867(T)	99.25	86.78	32.80
S. microflavus NBRC 13062(T)	99.18	89.46	39.00
S. badius JCM 4350(T)	99.18	86.76	32.90

Table S3. The analysis of Ahn75 genome by Type (Strain) Genome Server.

**Subject strain**	**dDDH (d0, in %)**	**C. I. (d0, in %)**	**dDDH (d4, in %)**	**C.I. (d4, in %)**	**dDDH (d6, in %)**	**C.I. (d6, in %)**	**G+C content difference (in %)**
S. rhizosphaericola 1AS2c	94.8	[92.5 – 96.5]	93.8	[92.0 – 95.3]	96.5	[94.9 – 97.6]	0.09
S. cavourensis JCM 4298	80.8	[76.9 – 84.2]	59.1	[56.3 – 61.8]	79.1	[75.6 – 82.1]	0.14
S. bacillaris ATCC 15855	74.2	[70.2 – 77.8]	60.7	[57.9 – 63.5]	73.9	[70.4 – 77.1]	0.37
S. cyaneofuscatus NRRL B-2570	58.8	[55.1 – 62.3]	40.1	[37.6 – 42.6]	54.8	[51.7 – 57.9]	0.77
S. microflavus JCM 4496	57.2	[53.6 – 60.7]	39	[36.5 – 41.5]	53.2	[50.1 – 56.2]	1.09
S. microflavus NBRC 13062	56.5	[52.9 – 60.0]	39	[36.5 – 41.5]	52.6	[49.5 – 55.7]	1.15
S. arboris TRM68085	53.8	[50.3 – 57.2]	40.2	[37.7 – 42.7]	50.8	[47.8 – 53.9]	0.9
S. setonii JCM 4516	48.4	[45.0 – 51.8]	33.2	[30.8 – 35.7]	44.2	[41.2 – 47.2]	0.45
S. globisporus JCM 4378	46	[42.7 – 49.5]	32.8	[30.4 – 35.3]	42.3	[39.3 – 45.3]	0.88
S. badius JCM 4350	45.5	[42.1 – 48.9]	32.9	[30.4 – 35.4]	41.9	[38.9 – 44.9]	0.65
S. durocortorensis RHZ10	43.4	[40.0 – 46.8]	33.6	[31.2 – 36.1]	40.4	[37.5 – 43.5]	0.63

Table S4. Gene clusters involved in the biosynthesis of secondary metabolites identified in the Ahn75 genome by antiSMASH.

Cluster	Type	Length （bp）	Most similar known biosynthetic gene cluster	Similarity	MIBiG BGC-ID
1	betalactone	27806	Divergolide	6%	BGC0001119
2	Butyrolactone	10951	Coelimycin	16%	BGC0000038
3	Butyrolactone-Ectoine	15157	Showdomycin	47%	BGC0001778
4	Nrps	58283	Valinomycin	22%	BGC0000453
5	Nrps	60582	Salinomycin	14%	BGC0000144
6	Nrps	46878	Griseoviridin	8%	BGC0000459
7	Nrps	37081	Malacidin	7%	BGC0001448/
8	Nrps	67376	Phosphonoglycans	3%	BGC0000806
9	Nrps	34256	Daptomycin	3%	BGC0000336
10	Nrps	25780	-	-	-
11	Nrps-like	43975	Bottromycin A2	39%	BGC0000469
12	Nrps-like, Arylpolyene	30267	WS9326	5%	BGC0001297
13	Nrps-T1pks	74299	Diisonitrile antibiotic SF2768	66%	BGC0001574
14	Nrps-TransAT-T1pks	52966	Cosmomycin D	5%	BGC0001074
15	Bacteriocin-Nrps-T1pks	50866	SGR PTMs	100%	BGC0001043
16	Siderophore	11779	Desferrioxamine B	100%	BGC0000941
17	Siderophore	54807	Griseobactin	94%	BGC0000368
18	Siderophore	49217	Coelichelin	81%	BGC0000325
19	Siderophore	14748	Ficellomycin	3%	BGC0001593
20	T1pks	29276	Bafilomycin	83%	BGC0000028
21	T1pks	24984	Bafilomycin	38%	BGC0000028
22	T1pks	11389	Salinomycin	14%	BGC0000144
23	T1pks	32285	Brasilinolide	5%	BGC0001381
24	T2pks	32180	Nonactin	78%	BGC0000252
25	T2pks	31610	Auricin	44%	BGC0000201
26	T3pks	41053	Alkylresorcinol	100%	BGC0000282
27	T3pks	41119	Herboxidiene	6%	BGC0001065
28	Terpene	25609	Isorenieratene	100%	BGC0000664
29	Terpene	26574	Hopene	69%	BGC0000663
30	Terpene	20516	Steffimycin	19%	BGC0000273
31	Terpene	27991	Stambomycin	8%	BGC0000151
32	Terpene	21119	-	-	-
33	Bacteriocin	11434	-	-	-
34	Lanthipeptide	14011	AmfS	100%	BGC0000496
35	Lanthipeptide	23117	-	-	-
36	Lassopeptide(ripp)	22770	Keywimysin	100%	BGC0001634
37	LAP, thiopeptide,	45914	Lactazole	22%	BGC0000606
38	LAP, thiopeptide	32602	-	-	
39	Ectoine	10399	Ectoine	100%	BGC0000853
40	Melanin	10465	Melanin	100%	BGC0000911

Table S5. Genes related to plant growth promotion in the Ahn75 genome.

gene	Gene product	Function
*des*B cluster	Desferrioxamine B	Siderophore synthesis
*dhb-gr*i cluster	Griseobactin	
*cch*H cluster	Coelichelin	
Ficellomycin gene cluster	Ficellomycin	
*nif*U	nitrogen fixation protein NifU	Nitrogen utilization
*gln*B	nitrogen regulatory protein P-II 1	
*moe*A	molybdenum cofactor biosynthesis protein MoeA	
*moa*D	molybdenum cofactor biosynthesis protein MoaD	
nir	nitrite reductase	
*pst*A	phosphate ABC transporter permease PstA	Phosphate utilization
*pst*B	phosphate import ATP-binding protein PstB	
*pst*C	phosphate ABC transporter, inner membrane subunit PstC	
*pst*S	phosphate ABC transporter substrate-binding protein PstS	
*trk*A	potassium transporter TrkA	Potassium utilization
*ktr*B	potassium transporter KtrB	
*kdp*FABC	potassium-transporting ATPase subunit A-C	
*dha*S	indol 3-acet-aldehyde dehydrogenase	
*trp*C	indole-3-glycerol-phosphate synthase	
*yhc*X	Nitrilase	
phy	4-phytase	Phytase synthesis
*tps*A	alpha,alpha-trehalose-phosphate synthase	Trehalose synthesis
*tre*PP	trehalose 6-phosphate phosphorylase	
*tre*Z	malto-oligosyltrehalose trehalohydrolase	
*als*S	Acetolactate synthase	3-hydroxy-2-butanone synthesis
*spe*E	Spermidine synthase	Spermidine synthesis
*spe*B	Agmatinase	

A

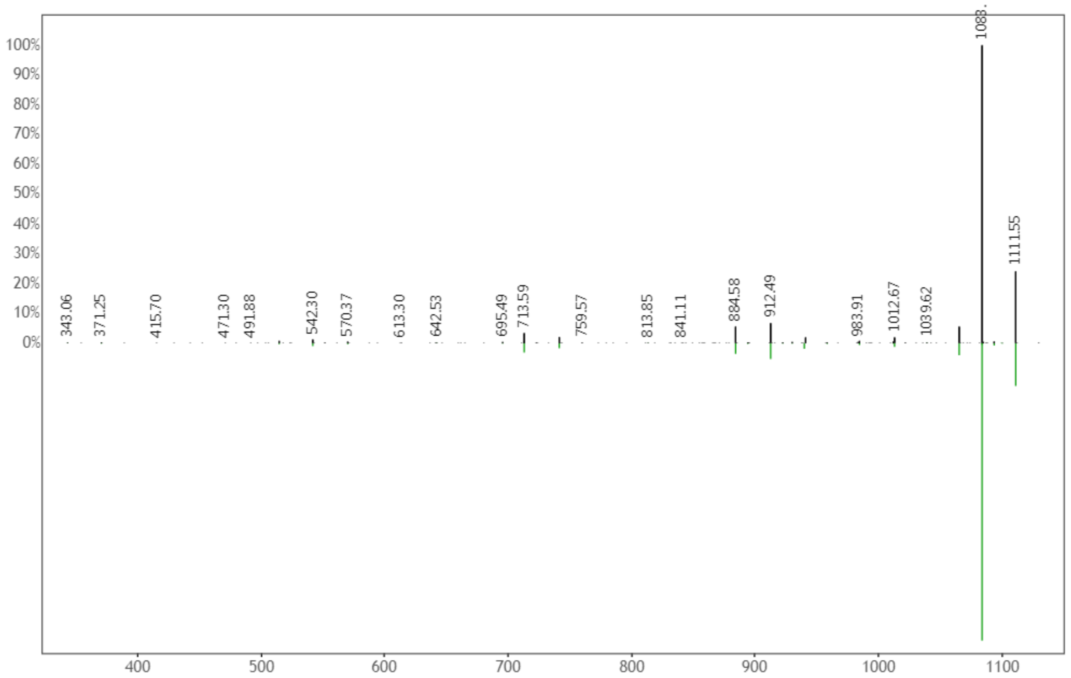
100%
90%
80%
70%
60%
50%
40%
30%
20%
10%
0%
1083.
1111.55
343.06
371.25
415.70
471.30
491.88
542.30
570.37
613.30
642.53
695.49
713.59
759.57
813.85
841.11
884.58
912.49
983.91
1012.67
1039.62
400
500
600
700
800
900
1000
1100

B

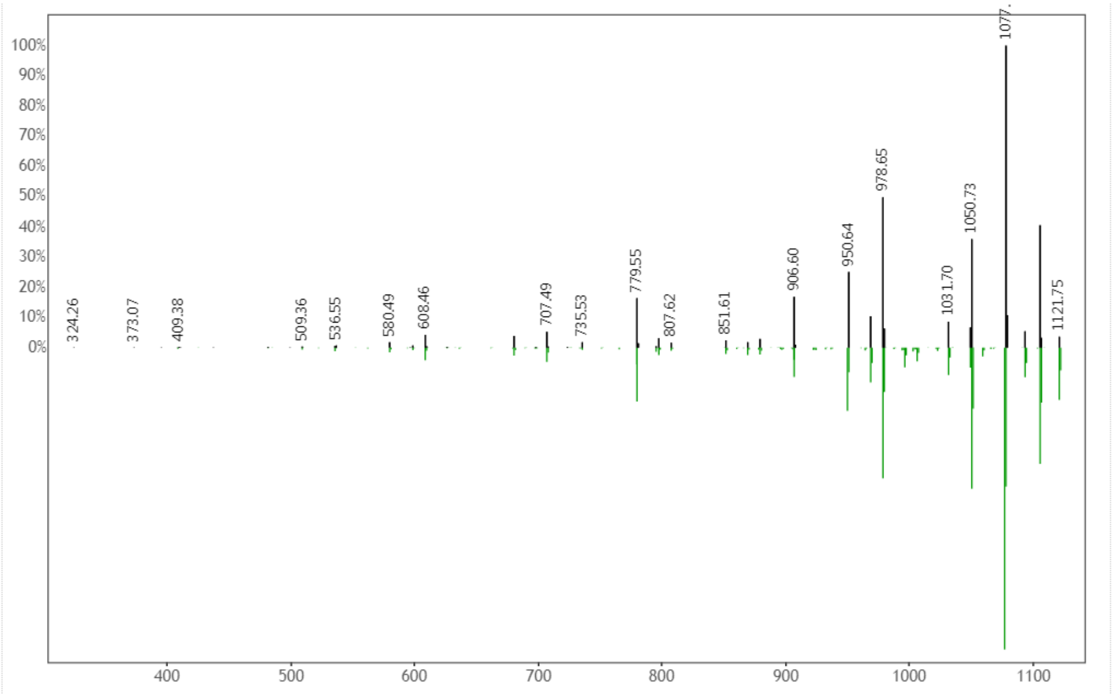
100%
90%
80%
70%
60%
50%
40%
30%
20%
10%
0%
1077.
978.65
1050.73
950.64
906.60
779.55
1031.70
1121.75
324.26
373.07
409.38
509.36
536.55
580.49
608.46
707.49
735.53
807.62
851.61
400
500
600
700
800
900
1000
1100

C

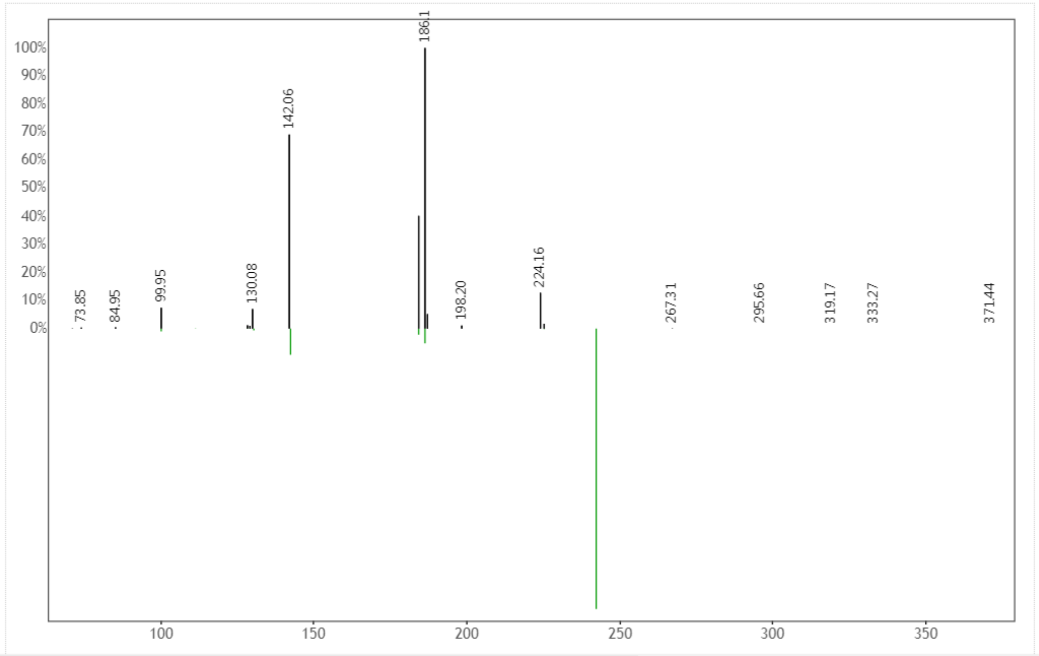

D

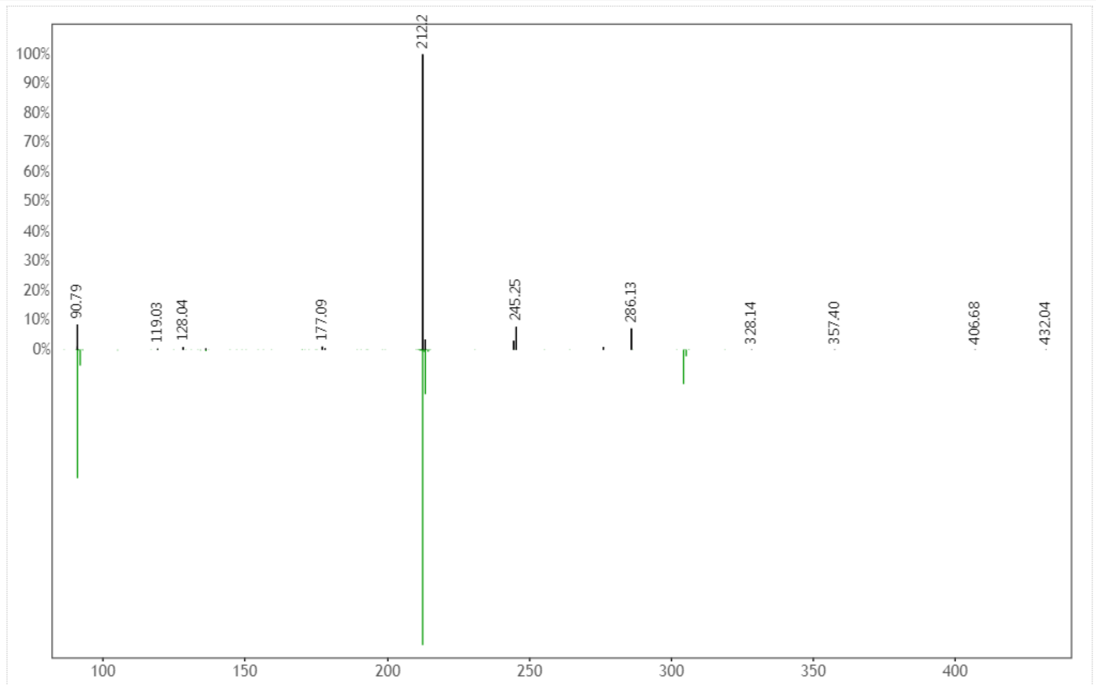

Fig. S1. Mirror-match of compounds with (A, B) valinomycin, (C) tetrabutylammonium and (D) benzalkonium chloride (C12) from the MASST GNPS database. (A) library class: Bronze; cosine-score: 0.99; shared peaks: 36; mass-diff: 0.01; specMZ: 1128.67; libMZ: 1128.66. **(B)** library class: Gold; cosine-score: 0.91; shared peaks: 36; mass-diff: 0.00; specMZ: 1149.60; libMZ:

1149.60. **(C)** library class: Bronze; cosine-score: 0.93; shared peaks: 8; mass-diff: 0.00; specMZ: 242.28; libMZ: 242.28. **(D)** library class: Bronze; cosine-score: 0.84; shared peaks: 7; mass-diff: 0.01; specMZ: 304.30; libMZ: 304.29.
